# Trends in diabetic ketoacidosis in Victoria, Australia 2002–2016

**DOI:** 10.1186/s12902-024-01650-0

**Published:** 2024-07-29

**Authors:** Hanna C. Jones, Katerina V. Kiburg, Melissa H. Lee, David N. O’Neal, Richard J. MacIsaac

**Affiliations:** 1 Department of Endocrinology & Diabetes, St Vincent’s Hospital Melbourne, 41 Victoria Parade, Fitzroy, VIC 3065 Australia; 2 Department of Medicine, University of Melbourne, Fitzroy, VIC 3065 Australia; 3 Australian Centre for Accelerating Diabetes Innovations, University of Melbourne, Parkville, VIC 3065 Australia

**Keywords:** Diabetic ketoacidosis, Type 1 diabetes, Type 2 diabetes, SGLT2 inhibitors

## Abstract

**Background:**

International longitudinal studies have indicated an increasing incidence of diabetic ketoacidosis (DKA). We aim to examine the incident trends, demographic differences, length of stay and mortality for DKA in adults with type 1 diabetes (T1D) and type 2 diabetes (T2D) in Victoria, Australia from 2002 to 2016.

**Methods:**

Age and sex adjusted incident trends, length of stay and mortality for DKA was retrospectively obtained using the Victorian Admitted Episode Dataset between 2002 and 2016. Data for adults with T1D and T2D was obtained from the National Diabetes Services Scheme (NDSS). Joinpoint regression analysis was used to identify changes in linear trends that were described as average annual percentage change (AAPC).

**Results:**

There were 23,628 DKA presentations in Victoria between 2002 and 2016. For T1D there was an increase in DKA presentations (AAPC + 6.8%) from 2003 to 2016 and for T2D there was a decline from 2003 to 2011 (APC − 3.5%), increase from 2011 to 2014 (APC + 38.5%), and a decrease from 2014 to 2016 (APC − 20.9%). Length of stay was longer for people with T2D than T1D (*P* < 0.001) and the mortality rate was 0.51% for the study period.

**Conclusions:**

DKA rates increased for T2D from 2011 to 2014 which correlates with the introduction of sodium glucose-linked transport protein 2 inhibitors. However, the aetiology for the observed increase in T1D from 2002 to 2016 remains unknown.

## Background

Diabetes affects a significant proportion of the Australian population, with one in twenty (5.3%) having a diagnosis of diabetes in 2020–2021 of which 11% have type 1 diabetes (T1D) and 85.5% have type 2 diabetes (T2D) [1]. A major consequence of diabetes is Diabetic Ketoacidosis (DKA) which is a largely preventable and treatable condition, but can result in significant morbidity, prolonged hospital stays and at times can be fatal [2].

DKA is a medical emergency characterised by hyperglycaemia, ketonaemia, and metabolic acidosis [2]. While the condition is usually associated with younger individuals with uncontrolled or undiagnosed T1D, it can still occur in people with T2D. In the United Kingdom (UK), one in five DKA admissions are reported to occur in patients with T2D [3].

Longitudinal studies in the United States (US) and the UK have demonstrated an increasing incidence of DKA for both T1D and T2D [3, 4]. Of note, the increase in DKA presentations antedated the coronavirus (COVID-19) pandemic and has occurred without any recent changes to the diagnostic criteria for DKA by the American Diabetes Association or The Joint British Diabetes Societies for Inpatient Care. Reassuringly however, a simultaneous decrease in mortality with DKA presentations has been reported [3, 4].

Additionally, a recent Australian study looking at incidence trends of hospitalisations for diabetes related complications, observed an increase in hospitalisation for hyperglycaemia, encompassing both DKA and hyperosmolar hyperglycaemic state (HHS), for adults with T1D and T2D from 2010 to 2019 [5].

However, a detailed analysis specifically related to DKA incidence trends by diabetes type has not been completed in Australia. This is particularly relevant as from the early 2000’s there have been changes in diabetes management including oral hypoglycaemic agents such as sodium glucose-linked transport protein 2 (SGLT2) inhibitors, increased uptake of insulin pump therapy, and access to blood ketone monitoring which may impact DKA risk specifically and provide insights for prevention strategies for this condition [6, 7].

In recognition that factors contributing to DKA presentations in adults may differ significantly from those in children and adolescents and that the COVID-19 pandemic had a major impact on DKA presentation rates, morbidity, and mortality we have restricted this analysis to adults and a timeframe antedating the pandemic [8]. We aimed to conduct a detailed retrospective examination of age and sex adjusted incident trends, length of stay and mortality specific to DKA admissions in Australian T1D and T2D adults living in the state of Victoria in the pre-COVID era between 2002 and 2016.

## Methods

### Data sources

Hospital discharge data and mortality records from the Victorian Admitted Episode Dataset (VAED) was obtained between 2002 and 2016, with 2002 being the reference year. Diagnostic information was coded according to the International Statistical Classification of Diseases and Related Health Problems, 10th Revision, Australian Modification (ICD-10-AM) [9]. Data were obtained through the National Diabetes Services Scheme (NDSS), which distinguishes between T1D and T2D and captures 80–90% of Australians with known diabetes [10].

### Definitions

As per the NDSS, T1D is defined as autoimmune destruction of beta cells of the pancreas resulting in decreased insulin production whereas T2D is defined as decreased insulin production and/or insulin resistance [10]. However, it should be noted that the type of diabetes coded by the NDSS is supplied by a diabetes related specialist without the need to provide any supportive information.

We identified incident cases of DKA using ICD-10-AM codes, where a DKA event was diagnosed as a medical issue during admission. The specific codes were as follows: E10.1X and E11.1X, that contained the term “ketoacidosis”. DKA events identified were divided into “first admission”, a patient’s first DKA admission in the study period, and “any admission”, where any subsequent DKA event was included. Those less than 21 years in age were excluded.

Of the identified cases, diabetes type, sex, and age-group were obtained. Diabetes type was characterised as either T1D or T2D and the VAED contains a Charlson Comorbidity Index (CCI) score for each patient admission [11].

DKA events were further analysed by inpatient length of stay and in hospital mortality where the primary cause of death was DKA. Where appropriate, the above outcomes were adjusted for a patient’s CCI score.

### Statistical analysis

Baseline characteristics were compared for patients with T1D and T2D using the chi-squared test for categorical variables and continuous variables were compared using an ANOVA test. Standardised rates were calculated by diabetes type observed in the NDSS population and expressed per 10,000 adults with 95% confidence intervals (using the STATA command *dstdize*). The total number of admissions for DKA and the diabetes status of patients was obtained through ICD-10-AM codes. Incident rate ratios were calculated and stratified by diabetes status using poisson regression models adjusted by age group and sex with an offset variable included to account for changes in the prevalence of diabetes over the observational period of the study using annualised NDDS data.

From the above we calculated incident rate ratios (relative to 2002) for DKA per year stratified by the two groups of patients according to their diabetes status. These changes were then analyzed by Joinpoint regression (version 4·7·0·0, Statistical Methodology and Applications Branch and Data Modeling Branch, Surveillance Research Program, National Cancer Institute) [12]. Beginning with one straight overall line for the time period, permutation tests were then used to identify points where linear trends changed significantly (*p* < 0·05) in either direction or magnitude. Then up to 3 joinpoints were added to the model to identify significant changes in the slope. Each trend segment was described by an annual percentage change (APC) and the trend for the entire study period (2002–2016) described by the average annual percentage change (AAPC), a summary measure of the trend accounting for each trend segment.

Analysis was done using STATA version 15·1 (StataCorp, College Station, TX). This study was approved by the St Vincent’s Hospital Melbourne Human Research Ethics Committee (HREC/18/SVHM/146).

## Results

### Total admissions

The total number of DKA presentations in Victoria between 2002 and 2016 was 23,628 (14,468 T1D and 9,160 T2D) (Table 1). There were 12,562 presentations for “first DKA admission” (5,083 T1D and 7,479 T2D) and 23,628 presentations for “any DKA admission” (14,468 T1D and 9,160 T2D), (Table 1). When standardised for age, sex and prevalence there was a higher incidence of DKA for T1D compared to T2D, averaging 43.24 per 1,000 presentations for T1D compared to 3.17 per 1,000 presentations for adults with T2D from 2002 to 2016.

**Table 1:** Patient characteristics by admission type

Patient characteristics						
	First admission			Any admission		
	T1D	T2D	*P* value	T1D	T2D	*P* value
Total (n)	5,083	7,479		14,468	9,160	
Sex						
Male	2,618 (51.5%)	4,198 (56.1%)	< 0.001	6,705 (46.3%)	5,093 (55.6%)	< 0.01
Female	2,465 (48.5%)	3,281 (43.9%)		7,763 (53.7%)	4,067 (44.4%)	
Age groups (years)						
21–29	2,273 (44.7%)	90 (1.2%)	< 0.001	6,876 (47.5%)	151 (1.6%)	< 0.001
30–39	976 (19.2%)	200 (2.7%)		3,065 (21.2%)	284 (3.1%)	
40–49	771 (15.2%)	540 (7.2%)		2,104 (14.5%)	728 (7.9%)	
50–59	461 (9.1%)	1,042 (13.9%)		1,180 (8.2%)	1,359 (14.8%)	
60–69	309 (6.1%)	1,675 (22.4%)		619 (4.3%)	2,060 (22.5%)	
70–79	183 (3.6%)	2,056 (27.5%)		390 (2.7%)	2,402 (26.2%)	
80+	110 (2.2%)	1,876 (25.1%)		234 (1.6%)	2,176 (23.8%)	
Length of stay (days)	4.9 (8.0)	13.4 (17.6)	< 0.001	4.7 (8.5)	13.1 (17.3)	< 0.001
**Deaths due to DKA**						
				**T1D**	**T2D**	***P*** **value**
Total (n)				100 (0.69%)	22 (0.24%) **Total** = 122 (0.51%)	
				**Alive**	**Dead**	
Age group (years)						
21–29				2,564 (21.7%)	86 (1.0%)	< 0.001
30–39				1,518 (12.9%)	186 (2.2%)	
40–49				1,586 (13.4%)	374 (4.4%)	
50–59				1,607 (13.6%)	761 (9.0%)	
60–69				1,782 (15.1%)	1,446 (17.1%)	
70–79				1,596 (13.5%)	2,407 (28.4%)	
80+				1,140 (9.7%)	3,216 (37.9%)	
Total (n)				11,793	8,476	

The main reason for admission was coded as DKA by a patient’s treating medical team. Causes for admission other than DKA include sepsis, pneumonia, acute kidney failure and congestive heart failure (Table 2).

**Table 2:** Top 10 reasons for admission for patients admitted to victorian hospitals, Australia with DKA. Patients were included in the study if their admission was associated with a ICD-10 code E10.1X or E11.1X that included the term ketoacidosis

First diagnosis	Frequency	Percent	Cumulative
E10.11 - Type 1 diabetes mellitus with ketoacidosis, without coma	13,143	38.8	38.8
E11.11 - Type 2 diabetes mellitus with ketoacidosis, without coma	1,964	5.8	44.6
E11.01 - Type 2 diabetes mellitus with hyperosmolarity without nonketotic hyperglycaemic-hyperosmolar coma [NKHHC]	1,442	4.3	48.8
A419 - Sepsis, unspecified	664	2.0	50.8
E11.02 - Type 2 diabetes mellitus with hyperosmolarity with coma	656	1.9	52.7
J189 - Pneumonia, unspecified	656	1.9	54.6
N179 - Acute kidney failure, unspecified	626	1.9	56.5
Z509 - Care involving use of rehabilitation procedure, unspecified	454	1.3	57.8
I500 - Congestive heart failure	451	1.3	59.2
E10.15 - Type 1 diabetes mellitus with ketoacidosis, with lactic acidosis, without coma	387	1.1	61.5

### Sex distribution

There was a higher proportion of males presenting with DKA for ‘first admission’ T1D (51.5%), ‘first admission’ T2D (56.1%) and ‘any admission’ T2D (55.6%) (Table 1). However, there was a higher proportion of females presenting with DKA for T1D ‘any admission’ (53.7%) (Table 1).

### Age

**Fig. 1:**
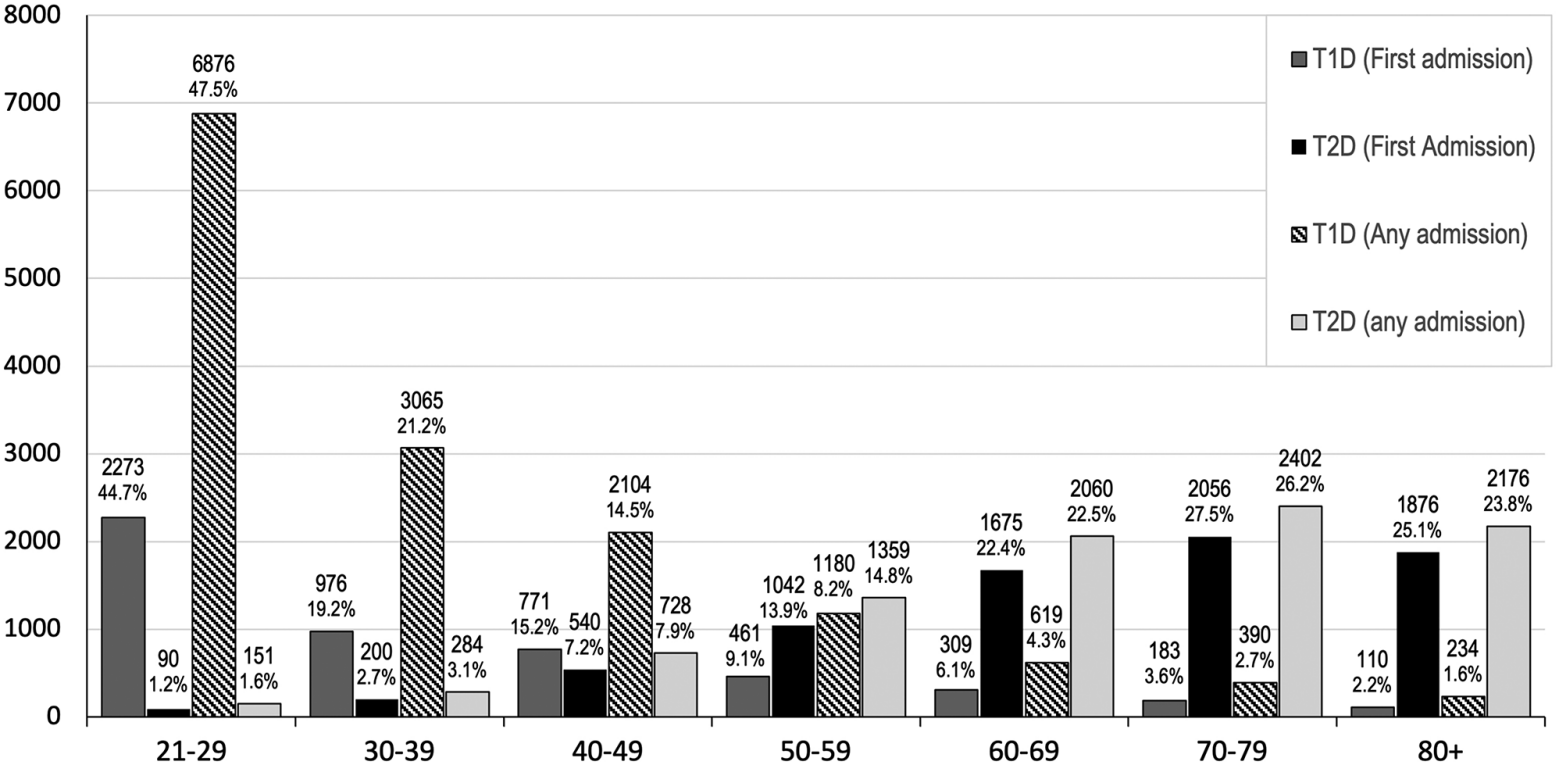
DKA rates by age-group

DKA in adults with T1D was more common in younger age groups and DKA rates decreased with increasing age with the 21–29-year-old group having the highest rate of DKA for both ‘first admission’ (2,273, 44.7%) and ‘any admission’ (6,876, 47.5%) (Table 1; Fig. 1). DKA in people with T2D was more common in older age groups and DKA rates increased as age increased with the 70–79-year-old group having the highest rate of DKA for both ‘first admission’ (2,056, 27.5%) and ‘any admission’ (2,402, 26.2%) (Table 1; Fig. 1). Both T1D and T2D had a higher CCI for adults aged 60 years or older compared to those less than 60 years (*P* < 0.001).

### Trends

**Fig. 2:**
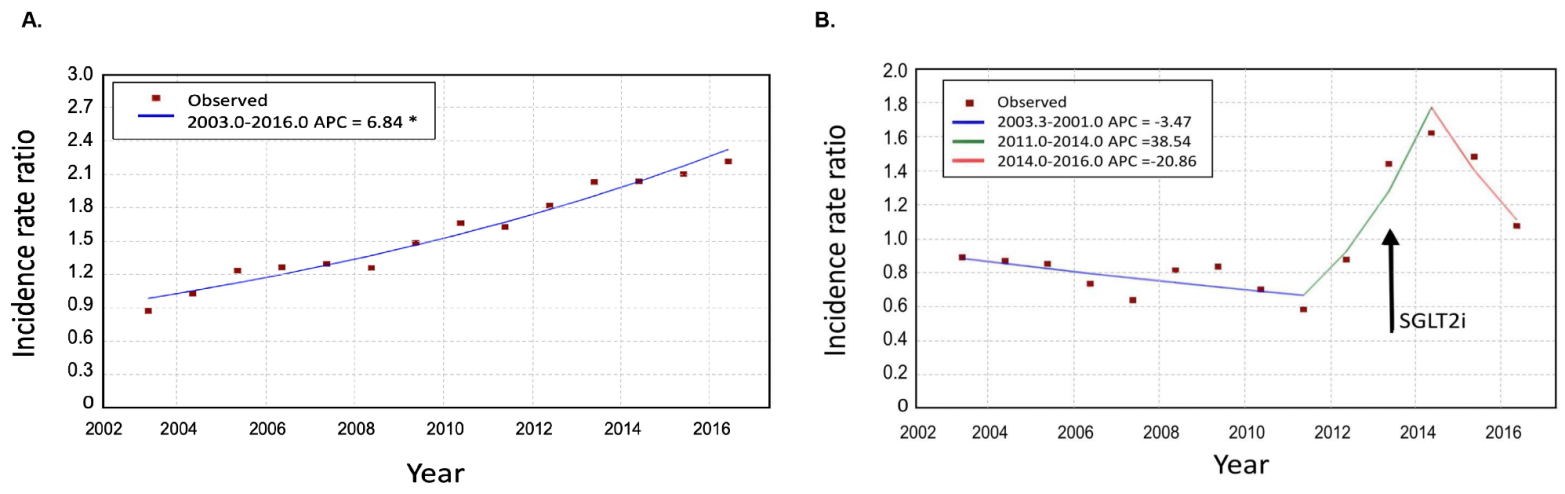
T1D DKA (panel A) and T2D DKA (panel B) presentation rates 2003-2016. *Indicates that the Annual Percentage Change (APC) is significantly different from zero at the alpha = 0.05 level. Arrow in panel B represents the year that SGLT2 inhibitors were introduced into the Australian market

There was an almost linear trend observed for DKA presentation rates for adults with T1D including 25.6 per 1,000 persons in 2003 compared to 67.7 per 1,000 persons in 2016, + 6.8 AAPC increase (Table 3; Fig. 2A). In contrast, DKA presentation rates in adults with T2D declined from 2003 to 2011, (2.4 to 2.2 per 1,000 persons, -3.5% APC), increased from 2011 to 2014 (5.8 per 1,000 persons, + 38.5% APC), and appeared to decrease from 2014 to 2016 (4.0 per 1,000 persons, -20.9% APC), although this downward trend was only based on admission rates from 2015 to 2016 (Table 3; Fig. 2B).

**Table 3:** Average annual percentage change (AAPC) and annual percent change (APC) for DKA by diabetes type between 2003 and 2016

	Overall trend* (2003–2016)	Initial trend*		Subsequent trends*		Subsequent trends*	
Diabetes Type	AAPC (95% CI)	Years	APC (95% CI)	Years	APC (95% CI)	Years	APC (95% CI)
Type 1 Diabetes	6.8 (5.9, 7.8)	-	-	-	-	-	-
Type 2 Diabetes	1.8 (-5.7, 9.8)	2003–2011	-3.5 (-7.2, 0.4)	2011–2014	38.5 (-0.7, 93.3)	2014–2016	-20.9 (-42.7, 9.3)

*Adjusted for age and sex

### Length of stay

The length of stay was longer for people with T2D than T1D admitted with DKA for ‘first admission’ (13.4 vs. 4.9 days, respectively, *P* < 0.001) and ‘any admission’ (13.1 vs. 4.7 days, respectively, *P* < 0.001) (Table 1). Adults with T2D had a higher proportion of comorbidities for all components of the CCI compared to adults with T1D (*P* < 0.001).

The overall length of stay and the above differences between T1D and T2D remained stable during the study period for both T1D and T2D.

### Mortality

The total number of deaths from 2003 to 2016 was 122 (0.51%). This included 100 (0.69%) deaths in people with T1D and 22 (0.24%) deaths in people with T2D (Table 1). For adults aged 65 years or older there was a total of 23 deaths including 9 deaths for people with T1D and 14 deaths for people with T2D. The small number of deaths precluded any further analysis of this outcome.

## Discussion

State-wide data from Victoria, Australia (VAED) indicated an increase in the rate of DKA presentations between 2003 and 2016. The increase in DKA rates, at least for T1D, is consistent with international studies of similar time periods [4, 13] and the aetiology is likely multifactorial. Precipitants include acute intercurrent illness, non-adherence to insulin, failure to detect and respond to rising glucose and ketone levels, insulin pump failure, lack of access to medical care, and insufficient education [13, 14]. Beyond 2020, the rates of DKA may be influenced by the COVID-19 pandemic which has been identified as a risk factor for developing DKA with increased severity and a higher mortality [8, 15].

Increased DKA presentations may also be attributed to increased recognition of DKA. Increased testing of ketone levels at home may encourage earlier hospital presentations with DKA. Studies have suggested that self-monitoring of ketones has led to fewer hospital admissions and shorter hospitalisation times [16]. However, ketone testing strips are not financially subsidised in Australia and a recent audit of our clinics revealed 30% of attendees did not have in-date ketone testing strips at home [17]. Similarly, Continuous Glucose Monitoring (CGM) use may also enable people with T1D to be more aware of an impending hyperglycaemic emergency however is it unlikely that CGM impacted trends in this study as utilisation rates were < 5% in Victoria prior to 2022 [7, 18].

A previous study demonstrated an increase in hospitalisations for hyperglycaemia, defined as including both DKA and HHS, from 2010 to 2019 for Australian adults with T1D and T2D [5]. In contrast, our study specifically analysed DKA trends in Victoria, included first and subsequent admission for DKA, and related these to CCI, length of stay and mortality over a 14-year period. By examining DKA specific trends, we hope to uncover strategies to reduce the incidence, morbidity and mortality of this preventable condition and allow for future comparisons of the impact advances in technology, such as CGM and closed-loop insulin delivery, have had on DKA hospital presentations.

The introduction of SGLT2 inhibitor use likely explains the increased DKA presentations observed from 2012 to 2014 in people with T2D. Prescriptions for SGLT2 inhibitors were first available in Australia in December 2013 and rose rapidly thereafter, coinciding with the observed increase in DKA presentations at this time [19]. In 2015, the Australian Therapeutic Goods Administration issued warnings regarding the association between SGLT2 inhibitors and increased risk of DKA [20], correlating with the decline in DKA presentation rates after 2015. In contrast, Morton et al. observed a steady increase in hyperglycaemic events for adults with T2D, however, this may be due to coding for all hyperglycaemic events rather than analysing for DKA alone [5]. It is worthy to note that we do not have data on how many patients were on SGLT2 inhibitors or how many presentations were consistent with euglycaemic DKA.

People with T1D and DKA were more likely to be younger, consistent with an increasing prevalence of DKA around the time of diagnosis of T1D, typically in individuals less than 25 years of age [21]. DKA hospitalisations have been found to peak in individuals with T1D around the age of 18–19 years, then decline following this [22]. Contrastingly, for T2D there were more DKA events in older age groups correlating with increased incidence of T2D, increased insulin dependence, a progressive reduction in β-cell function with diabetes duration, and an increase in co-morbidities with older adults [23]. This also correlates with an increased length of stay and a higher CCI observed in people with T2D compared to T1D. Of note, length of stay remained unchanged throughout the entire study period. It would be beneficial to see if increased access to healthcare professionals who specialised in DKA management would result in a reduced length of stay for people admitted to Victorian hospitals with DKA.

The low mortality rate of 0.5% is consistent with international data and indicates successful management of DKA during this study period [4]. While previous studies have noted that mortality rate in DKA tends to increase with age, we observed that majority of deaths (99 of 122 deaths) occurred in those less than 65 years old. A possible cause could be that admitted patients with T1D presented with a more severe metabolic disturbance than those with T2D, but the most likely explanation is that majority of DKA admissions in our data set were for people with T1D with approximately 90% of these admissions being for people aged < 60 years of age (Table 1; Fig. 1) [24]. Our reported mortality rate is based on deaths directly attributed to DKA which may be low as often the cause of death is attributed to the underlying precipitating illness which led to DKA, as discussed below.

Study limitations include that this was an observational and retrospective study. Additionally, data used was reliant on medical record documentation in the VAED where events may have been misclassified or missed, and data may be over-represented by individuals who presented multiple times. For example, one female participant with T1D presented over 100 times during the study period.

Furthermore, the classification of diabetes type was based on data obtained though the Australian NDSS. Biochemical parameters or diabetes specific autoantibodies were not used to confirm the accuracy of the diabetes type assigned to an individual and some individuals may have been misclassified, for example younger individuals being diagnosed with type 2 diabetes and older individuals being diagnosed with latent onset diabetes of adulthood (LADA). Socioeconomic status has also been linked with DKA, with those from a more disadvantaged background having an increased risk of admission for DKA, however our dataset did not allow for that type of analysis to be performed [25]. Our mortality data is based on deaths that were coded by a patient’s treating medical team as being directly attributed to DKA. There was no independent adjudication on the causes of death. Additionally, we did not include post-discharge mortality. Our data did not include intensive care unit admissions, clinical characteristics, or biochemical profiles such as the severity of DKA, nor did it link comorbidities at admission with length of stay. Finally, this dataset only includes data from Victoria and may not represent DKA presentations in other areas of Australia.

Strengths of this study include its long observational period of 14 years across large state-wide data under non-pandemic conditions which should represent the norm. Additionally, this study distinguishes DKA by T1D and T2D from NDSS data, sex, age groups, length of stay and mortality, and included adults with both new and established diagnoses of diabetes. The observations from this study are in keeping with comparable international and Australian data.

## Conclusions

In conclusion, from 2002 to 2016 we observed an increase in DKA events in Victorian adults with a mortality impacting younger people disproportionately. The aetiology for the increase observed is likely multifactorial and is comparable to studies in the UK and US. DKA is a preventable condition so enhancing education will be important in reducing the incidence, morbidity, and mortality of this condition. Finally, future research would be useful to observe how DKA trends changed with the COVID-19 pandemic, with CGM availability, and with advances in technology including continuous ketone sensors and closed-loop insulin delivery.

## Data Availability

The data that support the findings of this study are from the Victorian Admitted Episode Dataset (VAED) and the National Diabetes Service Scheme (NDSS), but restrictions apply to availability of these data, which were used under license for the current study, and so are not publicly available. Data are, however, available from the data custodians (VAED and NDSS) on request (pending ethical and data custodian approvals).
